# Montreal Cognitive Assessment for cognitive assessment in chronic kidney disease: a systematic review

**DOI:** 10.1590/2175-8239-JBN-2018-0086

**Published:** 2019-01-24

**Authors:** Thaís Malucelli Amatneeks, Amer Cavalheiro Hamdan

**Affiliations:** 1 Fundação Pró-Renal, Setor de Psicologia, Curitiba, PR, Brasil.; 2 Universidade Federal do Paraná, Departamento de Psicologia, Curitiba, PR, Brasil.

**Keywords:** Kidney Diseases, Mental Status and Dementia Tests, Cognitive Dysfunction., Nefropatias, Testes de Estado Mental e Demência, Disfunção Cognitiva

## Abstract

**Introduction:**

There is evidence in the literature that cognitive impairment is more prevalent in individuals with chronic kidney disease (CKD) than in the general population. The Montreal Cognitive Assessment (MoCA) is an instrument with a good application profile for cognitive evaluation of patients with CKD-like impairments. The objective of this study is to perform a systematic review of MoCA use in the context of CKD.

**Method:**

The keywords "Montreal Cognitive Assessment", "Kidney Disease" and "Chronic Kidney Disease" were used to search the databases. The inclusion criteria were: a) empirical articles; b) approach to cognitive impairment in CKD; c) papers in Portuguese and English.

**Results:**

The studies were mostly cross-sectional, published in medical journals, with research carried out mostly in Europe. About 45% of the studies had samples of less than 150 participants and variations in the prevalence of cognitive impairment were found ranging from 28.9% to 74.6%. The cutoff point for the identification of the impairment presented variation between the studies.

**Discussion:**

The results' analysis demonstrates the need for more complete studies on MoCA scoring and adaptation in its different versions. We recommend to the health professionals who will use the results in the clinical setting that the interpretation of the results be made in the light of studies more related to the context lived by the patients.

**Conclusions:**

The instrument is efficient to be used in several stages and treatment modalities of the disease. We point to the need to adapt a cut-off point for the instrument in the different translations of the instrument.

## Resumo

**Introdução:**

Há evidências na literatura de que o comprometimento cognitivo se apresenta com maior prevalência em indivíduos com Doença Renal Crônica (DRC) do que na população em geral. O Montreal Cognitive Assessment (MoCA) é um instrumento com bom perfil de aplicação para avaliação cognitiva de pacientes com comprometimentos similares aos da DRC. O objetivo deste estudo é realizar uma revisão sistemática sobre a aplicação do MoCA no contexto da DRC.

**Método:**

Foram utilizadas as palavras-chave "Montreal Cognitive Assessment", "Kidney Disease" e "Doença Renal Crônica" nas bases de dados. Os critérios de inclusão foram: a) artigos empíricos; b) abordagem do comprometimento cognitivo na DRC; c) trabalhos em língua portuguesa e inglesa.

**Resultados:**

Os estudos foram em sua maioria transversais, publicados em periódicos médicos, com pesquisas realizadas majoritariamente na Europa. Cerca de 45% das pesquisas tiveram amostras inferiores a 150 participantes e foram encontradas variações na prevalência de comprometimento cognitivo variando de 28,9% até 74,6%. O ponto de corte para identificação do comprometimento apresentou variação entre os estudos.

**Discussão:**

A análise de resultados demonstra a necessidade de estudos mais completos sobre pontuação e adaptação do MoCA em suas diferentes versões. Recomenda-se aos profissionais de saúde que utilizarão os resultados em âmbito clínico que a interpretação dos resultados seja realizada por estudos mais relacionados ao contexto vivido pelos pacientes.

**Conclusões:**

O instrumento demonstra-se eficiente para ser utilizado em diversos estágios e modalidades de tratamento da doença. Aponta-se a necessidade da adaptação de um ponto de corte para o instrumento nas diferentes traduções do instrumento.

## Introduction

There is evidence in the literature that cognitive impairment is more prevalent in individuals with chronic kidney disease (CKD) than in the general population, considering any of the disease stages.^1^^,^ 2 It is considered that CKD diseases themselves and comorbidities are risk factors for the cognitive impairment of the patient. In addition to systemic arterial hypertension and diabetes mellitus, the predominance of factors for vascular risk, reduction in glomerular filtration rate,^3^ uremic toxins, polypharmacy, immunoinflammatory processes, anemia, oxidative stress and Renal Replacement Therapy (RRT) may be responsible for affecting the cognition of these patients.^4^ Pathophysiological mechanisms that promote neurological impairment can cause chronic degenerative changes in both the kidneys and the brain.^3^

Identifying cognitive impairment enables us to adequately improve care concerning the patient's cognitive reality. It assists in patient orientation, encouragement in the choice of treatment, and in the involvement of family members and caregivers in clinical consultations. Knowing the cognitive aspects of the patient simplifies care and enables a better use of the information, in order to aid in treatment compliance,^5^ since cognitive dysfunction is associated with greater risks of death and lower compliance.^6^

The use of cognitive screenings provides objective evidence of moderate to severe cognitive impairment in up to 70% of patients with CKD. The changes found usually indicate a combination of neurodegenerative dementia, such as Alzheimer's disease and vascular dementia. Even in the absence of obvious neurological changes, cognitive impairment can be detected in CKD patients through the use of psychometric instruments, such as the Montreal Cognitive Assessment (MoCA).^3^

MoCA has been considered a superior instrument to the Mini Mental State Examination for the screening of cognitive impairment in several pathologies that involve damage to subcortical structures of the nervous system, such as Parkinson's disease and diabetes mellitus.^2^ MoCA is one of the screening tests, which can be used by any trained healthcare professional. It has been developed specifically for the screening of milder forms of cognitive impairment, and presents high sensitivity and specificity for the detection of mild cognitive impairment, with an average application time of 10 minutes. It covers important cognitive domains and has application versions in several languages.^7^

There are few studies on the use of MoCA in the context of CKD. Therefore, the objective of this study is to perform a systematic review on the application of MoCA for the cognition evaluation of chronic renal patients in the context of CKD.

## Method

According to the PRISMA^8^ protocol for conducting review methods, it is important to identify the Population (P), the Intervention (I), the comparison (C where relevant) and the outcomes one wishes to assess (O). In this case, we intended to investigate the MoCA instrument use to assess cognition (I), to evaluate its efficiency for the context (O). One of the authors selected and extracted the papers individually. During the month of October 2018, we searched for original papers indexed in PubMed, Scopus, LILACS, PePSIC and SciELO databases. We used the keywords "Montreal Cognitive Assessment", "Kidney Disease" and "Chronic Renal Disease" without restricting for year of publication. The complete electronic search strategy can be found in the Supplemental Material section of this paper. The inclusion criteria were: a) empirical articles; b) those addressing cognitive impairment in chronic kidney disease; c) studies published in Portuguese, English and Spanish. The exclusion criteria were: a) non-use of the MoCA instrument in the study; b) articles that were not about chronic kidney disease; c) literature review articles; d) studies in other languages. The exclusion criteria were used based on the goal this review, seeking to evaluate how the empirical studies in the chronic renal patient population use the instrument. The papers were selected after reading the summary and the study methods, checking whether they were adequate vis-à-vis the inclusion and exclusion criteria. Subsequently, the duplicate papers were removed, thus getting to the final database.

We extracted the papers by means of an ordered table, extracting the following data: 1) Source database, 2) Year of publication, 3) Journal, 4) Country of research, 5) Sample of renal treatment researched, 6) Objectives, 7) Methods, 8) Associated instruments, 9) Sample number, 10) Main findings, 11) Limitations presented and 12) Conclusion.

## Results

**Figure 1:**
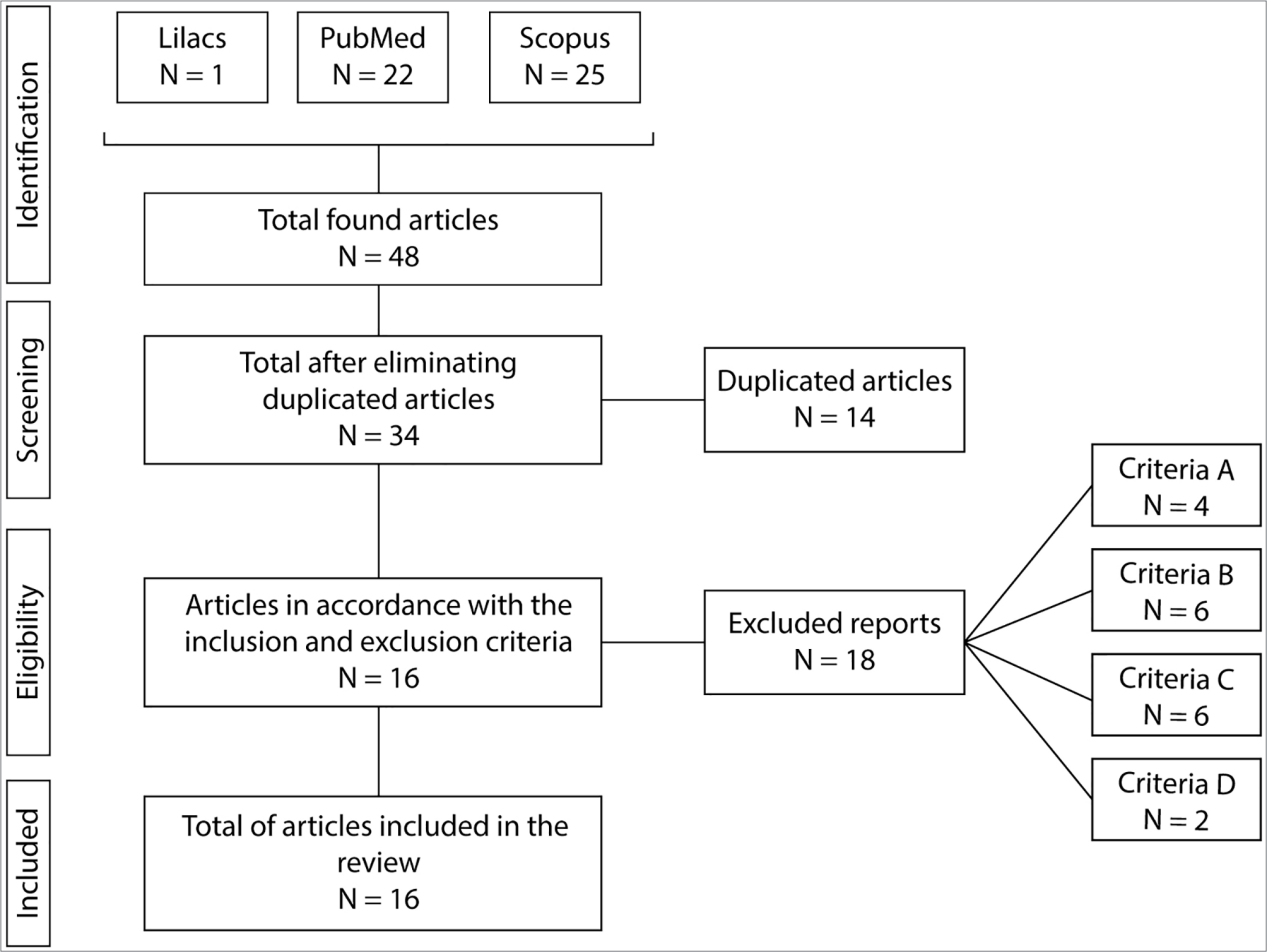
Selection of papers analyzed in this systematic review

The selection of the analyzed papers was carried out as exemplified in Figure 1. In total, we found 45 papers per search with the keywords in the databases. After removing the duplicate papers, we ended up with 34 papers to be evaluated according to the inclusion and exclusion criteria. Concerning the exclusion of papers, four of them were withdrawn as per the first criterion, since they did not use the MoCA as part of the methodology (criterion A); six papers were withdrawn because they did not deal with CKD (criterion B); seven papers were not empirical studies C), and two papers were only available in Chinese (criterion D). The final analysis involved 16 papers.

The year of publication of the 16 analyzed papers were concentrated between 2014 and 2018, six of them published in the year 2017, two in the years of 2015 and 2018 and three in the years of 2014 and 2016. Table 1 presents information about the country of the study, chronic kidney sample studied, study sample size, and journals in which the studies were published.

**Table 1:** Studies decribed by journal, country of origin, chronic renal patients' sample analyzed, sample size and study design

Studies	Journal	Country of origin	CKD sample	Sample size	Study design
Lee et al., 2018	Renal Failure	South Korea	HD	30 + 30 controls	Cross-sectional
Kim, Kang e Woo, 2018	Journal of Korean Medical Science	South Korea	HD	102	Cross-sectional
Otobe et al., 2017	Nephrology (Carlton)	Japan	Pre-dialysis;	120	Cross-sectional
Gupta et al., 2017	Nephron	United States	TX	157	Cross-sectional
Zheng et al., 2017	BMC Nephrology	China	PD	72	Cross-sectional
Iyasere, Okai e Brown, 2017	Clinical Kidney Journal	United Kingdom	Pre-dialysis; HD; PD	102	Longitudinal
Gupta et al., 2017	BMC Nephrology	United States	TX	226	Cross-sectional
Angermann et al., 2017	Clinical Science	Germany	HD	201	Cross-sectional
Paraizo et al., 2016	Jornal Brasileiro de Nefrologia	Brazil	Pre-dialysis	72	Cross-sectional
Foster et al., 2016	American Journal of Nephrology	Canada	Pre-dialysis;	385	Cross-sectional
Lambert et al., 2016	Nephrology (Carlton)	Australia	Pre-dialysis;		
HD; TX	155	Cross-sectional			
Shea et al., 2015	Peritoneal Dialysis International	China	PD	114	Longitudinal
Kang et al., 2015	Hemodialysis International	South Korea	HD	101	Cross-sectional
Tiffin-Richards et al., 2014	Plos One	Germany	HD	48 CKD + 42 controls	Cross-sectional
Palmer et al., 2014	American Journal of Nephrology	United States	CKD in initial stages	263	Cross-sectional
Nikić et al., 2014	BioMed Research International	Serbia	HD	86	Cross-sectional

HD = Hemodialysis; PD = Peritoneal dialysis; TX = Transplant

With regards to the country of origin, of Eastern countries stand out in the number of publications in this matter. The majority of the sample consisted of patients on hemodialysis. 41.1% of the papers had a sample of over 150 participants. The studies were published mostly in specific journals of nephrology.

Upon assessing the study methods, we found that only two of the studies were of a longitudinal nature.^9^^,^ 10 All the studies analyzed had a sample consisting of patients over 18 years of age. Table 2 summarizes demographic and clinical data, as well as the results concerning the MoCA application in the studies analyzed.

**Table 2:** Demographic, clinical and Montreal Cognitive Assessment instrument data in the analyzed studies

	Sample	Mean age (pd)	Gender (males)	< 12 years schooling	Prevalent baseline disease	Mean estimated GFR (sd)	Cutoff criterion	Prevalence of CI	Mean MoCA Score (sd)
Leeet al., 2018	HD	64.90 (7.88)	40.0%	-	-	-	Defined by age and education	-	20.35 (4.54)
Kim, Kang e Woo, 2018	HD	57.1 (12)	53.9%	-	Diabetic nephropathy (52.0%)	-	22	-	19.26 (7.78)
Otobe et al., 2017	Pre-dialysis	77.3 (6.8)	76.7%	43.3%	Hypertensive nephropathy (41.7%)	30.2 (12.5)	26	62.5%	24.4 (2.8)
Gupta et al., 2017	TX	55 (14.8)	57.0%	36.9%	-	50.3 (13.3)	26	30.0%	26.6 (2.9)
Zhenget al., 2017	DP	56.2 (16)	37.5%	40.3%	-	-	26	86.8%	21.7 (5.6)
Iyasere, Okai e Brown, 2017	DRC	72.5 (1.5)	63.9%	65.5%	Diabetic nephropathy (40.0%)	17 (0.9)	26	53.8%	25
	HD	68.9 (1.3)	70.7%	61.1%	Glomerulonephritis (26.8%)	-		63.3%	23
	DP	72.8 (1.6)	76.0%	100.0%	Diabetic nephropathy (63.9%)	-		64.3%	24
Gupta et al., 2017	TX	54 (13.4)	60.6%	57.5%	-	52 (21)	26	58.0%	
Angermann et al., 2017	HD	64.5	70.1%	39.2%	-		26	60.2%	24.14
Paraizo et al., 2016	Pre-dialysis	56.74 (7.63)	55.6%	-	-	-	24	73.6%	21.83 (4.16)
Foster et al., 2016	DRC 4 and 5	68	60.6%	-	-	19	24	61.0%	22.75
Lambert et al., 2016	Pre-dialysis	70	45.8%	54.2%	-	11.9 (4.7)	24	16.7%	22.07
	HD	70	52.0%	72.0%	-	-		48.0%	24.8
	DP	70.2	66.7%	63.0%	-	-		55.6%	23.12
	TX	58.5	61.5%	44.2%	-	58.3 (18.3)		19.2%	26.77
	Total	66	59.4%	56.8%	-	43.1 (26.7)		36.1%	25.23
Shea et al., 2015	PD	59 (15)	53.0%	44.7%	Diabetic nephropathy (31.6%)	-	21/22	28.9%	23 (5.3)
Kang et al., 2015	HD	57.3 (12.2)	53.9%	-	-	-	22	56.4%	19.04 (8.07)
Tiffin-Richards et al., 2014	HD	58.3 (13.9)	52.1%	-	Diabetic nephropathy (27.1%) Chronic glomerulonephritis (27.1%)	-	24	-	24.0 (4.0)
Palmer et al., 2014	CKD	60.5 (9.8)	40.7%	56.0%	-	75.3 (28)	-	-	19.27 (3.73)
Nikic, Andric e Stojimirovic, 2014	HD	60.9	69.7%	48.8%	-	-	26	75.6%	22.5

HD = Hemodialysis; TX = Transplant; PD = Peritoneal Dialysis; CKD = Chronic Kidney Patient; GFR = Glomerular Filtration Rate; CI = Cognitive Impairment; MoCA = Montreal Cognitive Assessment

Most of the studies had over 50% prevalence of cognitive impairment in their sample. As a criterion for the identification of cognitive impairment, the studies had different cutoff points, referring to different criteria for setting these values. Seven studies used the originally proposed cut-off point of 26 points, four used 24, and two other studies used the score of 22. Two studies did not specify the criteria used. Regarding Ktv values in dialysis patients, only four studies presented values, all of them above 1.2, as idealized by the health parameters. 

Only two studies presented the definition of what was understood by cognitive impairment, agreeing among themselves, explaining the term as an alteration in cognition in one or more domains, with preservation of functional and independent abilities, without prejudice to daily living activities.^2^^,^ 5 One of the studies does not define, but exemplifies that cognitive impairment would be a condition between expected cognitive decline for age and dementia.^11^ Table 3 presents data with reference to study objectives, methods used, and limitations presented by authors about the study.

**Table 3:** Objectives, methods, criteria and limitations of the studies

Authors	Objectives	Method	Limitations
Lee et al., 2018	Examine the cognitive function of patients in HD comparing two commonly used screenings to identify cognitive deficits.	Demographic and clinical data Laboratory tests Seoul Neuropsychological Screening Battery (SNSB) Geriatric Depression Scale MoCA MMSE	Relatively small sample; Limited clinical data for the control group; Cognitive abilities test immediately prior to dialysis.
Kim, Kang & Woo, 2018	To determine the relationship between psychosocial factors and QOL in patients with CKD undergoing hemodialysis.	Clinical data Laboratory tests WHOQOL-BREF Hospital Anxiety and Depression Scale Multidimensional Scale of Social Support Perceived MoCAPittsburg Sleep Quality Index Zarit Burden Interview	Selection bias in the choice of medical and psychosocial factors; Relatively small sample; Sample composed of a single center.
Gupta et al., 2018	To assess the advantage of cognition measured with standard screening tools on perceived cognition in transplant patients.	Demographic and clinical data MoCA Likert scale for perception of dementia	Limited results generalization due to sample demographic characteristics; Lack of detailed neuropsychological assessment; Need for validation of the Likert scale used.
Otobe et al., 2017	To assess the prevalence of CCL and the relationship between CCL and physical function in the elderly with pre-dialysis CKD.	Demographic and clinical data Laboratory tests Barthel Index MMSE MoCA Gait speed Manual gripping force Knee extensor muscle strain One-sided posture	MoCA use only to evaluate cognition; Non-use of control group of the same age; No evaluation of other influencing factors on cognition (such as depression, CKD duration); Exclusion of patients with probable dementia.
Zheng et al., 2017	Investigate Small Cerebral Vascular Diseases (DPVC) in patients on PD and determine the possible pathogenic mechanism of the disease and its functional alterations.	Demographic and clinical data Body mass index Laboratory tests Magnetic Resonance Imaging (DISCOVERY MR750; General Electric, Milwaukee, WI) Interviews MMSEMOCA	Relatively small sample; Confounding factors for MRI and cognitive alterations; Use of MOCA and MMST as a continuous variable rather than dichotomous.
Iyasere, Okai & Brown, 2017	To compare the cognitive tendencies between dialysis and CKD patients and, subsequently, between HD and PD patients.	Demographic and clinical data MoCA Patient Health Questionnaire-9 (PQH-9) MacArthur Competency Assessment Tool	Exclusion of patients with significant CI; Relatively small sample.
Gupta et al., 2017	To evaluate the prevalence of cognitive impairment in patients who are candidates for kidney transplantation.	Demographic and clinical data	Exclusion of patients with significant CI; Cross-sectional methodology prevented relevant longitudinal analyzes.
Angermann et al., 2017	To identify risk factors with a high impact on the pathogenesis of cognitive impairment and dementia in patients on HD, with a special focus on the role of vascular rigidity.	Demographic and clinical data MoCA Heart beats Blood pressure Pulse wave velocity	Exclusion of patients with significant CI; Use of only one cognitive evaluation tool; The method chosen for the measurement of PWV.
Paraizo et al., 2016	To determine the prevalence of CI in non-elderly patients with predialytic CKD; To identify neuropsychological tests that are easy to apply and interpret, for CI screening with MoCA-like performance.	Structured anamnesis Clinical questionnaire of depression MoCA Pfeffer Scale	*Not shown in the study.
Foster et al., 2016	To determine CKD prevalence and risk factors in patients with CKD stages 4 and 5, outside of RRT.	Demographic and clinical data Issues for assessing fragility MoCA	Lack of normative values in MoCA; Does not collect or evaluate laboratory serum values.
Lambert et al., 2016	To compare the extent of cognitive impairment and types of cognitive deficits in four groups of CKD patients.	Demographic and clinical data MoCA	Exclusion of patients with significant CI; Lack of record of factors that interfere with CI (depression); Lack of normative values in MoCA.
Shea et al., 2015	To investigate the prevalence of CKD in patients newly started in PD and the impact of CI in peritonitis related to dialysis.	Demographic and clinical data Laboratory tests MoCA	Lack of detailed cognitive history of the sample; Relatively small sample.
Kang et al., 2015	To identify the possible predictors of HRQOL among the clinical and psychosocial factors of patients in HD.	Demographic and clinical data Laboratory tests Hospital Anxiety and Depression Scale Multidimensional Scale of Perceived Social Support Pittsburgh Sleep Quality Index European Quality of Life Questionnaire 5 dimensions MoCA	Relatively small sample; Lack of collection of longitudinal questions about clinical, psychosocial and HRQOL factors; Conducting the study in a single center.
Tiffin-Richards et al., 2014	To evaluate MoCA as a short screening tool for CI in HD patients compared to a comprehensive cognitive test.	Clinical data MoCA Reverse digits Stroop Test Boston Appointment Test Spatial Perception and Object Scale Epworth Sleepiness Scale Brief Fatigue Inventory Hospital Anxiety and Depression Scale	Exclusion of patients with significant CI; Relatively small sample.
Palmer et al., 2014	To assess the relationship between mild renal disease and cognitive performance in the African-American population with DM2.	Demographic and clinical data Laboratory tests MoCA MMSERey's auditory-verbal learning test Stroop Test Verbal fluency for animals Code - WAIS III Laboratory tests	Difficulty in generalizing the results; Methodological questions about the study participants.
Nikic, Andric & Stojimirovic, 2014	To explore the effects of habitual coffee consumption and normal caffeine consumption on the cognitive function in patients under maintenance HD.	Demographic and clinical data Dietary questionnaire on habitual coffee consumption MoCA Beck Depression Inventory II Beck Anxiety Inventory FACIT Fatigue Scale Epworth Sleepiness Scale Athens Insomnia Scale Pittsburgh Sleep Quality Index	Relatively small sample; Lack of normative values in MoCA.

Legend: CKD = Chronic Kidney Disease; HD = Hemodialysis; PD = Peritoneal Dialysis; CI = Cognitive Impairment; PWV = Pulse Wave Velocity; RRT = Renal Replacement Therapy; HRQOL = Health-Related Quality of Life; DM2 = Diabetes Mellitus type 2; MoCA = Montreal Cognitive Assessment; MMSE = Mini Mental State Examination.

The studies are distributed heterogeneously in three main functions: a) estimate the prevalence of cognitive impairment in the population with CKD; b) use MoCA as a tool for cognitive measurement for intervention/exposure strategies; c) compare MoCA to other instruments for cognitive screening. The main findings and limitations of the study will be analyzed through these functions.

Regarding studies that sought to estimate the prevalence of cognitive impairment in the population with CKD,^2^^,^ 5 ^,^ 9 ^,^ 11 ^-^ 13 the main finding is the high prevalence of cognitive impairment found in the samples. We found out that age, education, basic diseases and being in renal replacement therapy may influence cognitive impairment;^5^^,^ 9 ^,^ 10 ^,^ 12 ^,^ 13 however, no significant differences were found between dialysis modalities in general scores.^9^ We also found that the etiology of cognitive impairment may not be entirely attributed to low rates of glomerular filtration^11^^,^ 12, demonstrating that albuminuria was associated with statistically significant worse performance.^14^ The results suggest that cognitive impairment in dialysis patients may not be fully reversible after transplantation.^12^

Since the possibly most influential limitation pointed out in more than one study, was the fact that MoCA does not have standardization values that help identify cognitive impairment in the CKD population. In addition, other important limitations involve methodological issues such as: exclusion of patients with significant cognitive impairment and non-measurement of characteristics that could influence cognitive impairment (such as serum values, depression, clinical data). Sample size and lack of a control group are also limitations, but seem to have less influence on the proposed objective.

Studies that used MoCA as a tool for cognitive measurement for intervention/exposure strategies^10^^,^ 11 ^,^ 15 ^-^ 18 found that cognition is an important factor to improve patients' clinical condition, even without being directly related to quality of life. They point out that the reduction in walking speed; arterial stiffness and lacunar infarction are important predictors of cognitive decline. Regarding the limitations presented in these studies that can influence the results, we stress the use of only one instrument to assess cognition and the non-assessment of other factors influencing cognitive impairment. Nonetheless, other factors considered less influential to their objectives are the exclusion of patients with probable dementia and the relatively small sample size, or only one center.

Finally, two studies comparing MoCA to other instruments for cognitive screening^2^^,^ 19 ^-^ 21 have pointed out that the instrument has important characteristics to be considered as a good evaluation tool in this population. MoCA demonstrates good levels of sensitivity and specificity, covering the main cognitive functions;^(2.21)^ among them, the executive functions, which play an important role in the cognitive performance of CKD patients. The instrument's short application time was also presented as an advantage of the instrument.^2^ The instrument is presented as an essential complement to the clinical practice, as it assists in the use of health care resources, with the objective of improving individual outcomes, and the possibility of longitudinal measurements due to the alternative versions available.^21^ The main limitations of this category were: a relatively small sample, lack of detailed neuropsychological evaluation, cognitive skills testing immediately prior to dialysis, and exclusion of patients with possible dementia.

Overall, the findings of the studies suggest the need for new longitudinal studies and larger samples. They also suggest that cognitive screenings are incorporated into routine clinical practice, using cognitive impairment data to plan patient education and compliance monitoring. In addition, the studies also point out the need for interventions and strategies to improve the cognition of patients in this context.

## Discussion

The objective of this study was to perform a systematic review on the application of MoCA in CKD. The results showed that MoCA is an effective tool for the cognitive assessment of patients at various stages of the disease and for the treatment modalities of CKD. However, there is no consensus in the literature regarding the best cutoff point for detecting the best sensitivity and specificity of the instrument. Although the instrument was developed in 2005, its use has become routine for this population only in the last five years. Recent use may be attributed to studies that demonstrate their superiority to Mini Mental State Examination in the identification of cognitive impairment.^2^

Its efficiency has been demonstrated due to the sensitivity and specificity of the instrument,^5^^,^ 21 coverage of the main cognitive domains and also the ease of application in the context.^2^ The instrument has been shown to be easily applicable and sensitive to capture the patients' degree of Impairment in the different stages of the disease, providing for the possibility of caring out longitudinal studies.^21^ From the point of view of the healthcare teams, it is effective, considering the time taken to be applied and instrument accessibility,^2^ considering costs and operating protocols.

The prevalence of studies involving patients in hemodialysis is because this population presents a higher risk factor for the development of cognitive impairment,^1^ worse quality of life,^22^ and requires stricter treatment compliance. Thus, it is important for healthcare professionals to identify cognitive impairment in order to seek interventions that are better suited to the patient.

It is paramount to collect clinical data and medical history of the patients when performing a cognitive evaluation study in chronic renal patients. Such data becomes fundamental, since cognitive dysfunction may be present even before the establishment of CKD, and the change is caused by the underlying diseases, comorbidities and/or diseases of the patient's history. In the same way that the kidney, when exposed to a high blood flow volume, may present lesions, the brain may be equally susceptible to these vascular damages and microvascular pathologies.^3^ Diabetes mellitus, another disease that is a risk factor for the development of CKD, also causes a certain level of impairment of some cognitive functions over the years of illness,^23^ with glycemic control being the cause of severe neurological injuries.^24^

The studies we analyzed did not present correlations of cognitive impairment on the different pathologies and laboratory tests, so it is interesting for new studies to try and find the differences between the cognitive profile of the patients with the different underlying diseases, comorbidities and clinical situation. Despite the lack of distinction between the factors presented previously, the studies found high rates of cognitive impairment in the sample, ranging from 28.9%^10^ to 74.6%.^2^ The lowest index was found in the study with patients on peritoneal dialysis, with prevalence of higher education; however, six other papers^5^^,^ 9 ^,^ 11 ^,^ 12 ^,^ 15 ^,^ 17 presented a prevalence above 58% of cognitive impairment in the sample.

Another of the limitations presented by the studies was related to the proposed methods vis-à-vis the study design and sample. Cross-sectional designs are most commonly preferred in these populations due to high mortality rates at treatment onset,^25^ hospitalization, and other factors that may compromise patient participation in the study. Studies bearing with larger samples, with greater potential for generalization, tend to overcome some of these limitations of the current cross-sectional samples. However, the need for longitudinal studies in this context is not ruled out.

The studies were hampered by the lack of standardization of scores to identify cognitive impairment. It is assumed that the estimate is easily manipulated by changing the cutoff value.^26^ This is confirmed when, in the same study, different prevalence rates of cognitive impairment were obtained in the population, from 61% with cutoff point of 24 to 75% with cut-off point of 26.^5^ value proposed by test developers.^7^

Although the study carried out by Tiffin-Richards et al. (21), included in this review, presented 24 points as the ideal cut-off score for the chronic renal population analyzed, the study presents several methodological limitations that make it difficult to generalize. The small sample and sociodemographic issues presented, such as high schooling, do not cover the reality of renal patients in many contexts, especially in developing countries and the poorest populations, where the rate of CKD patients increases.^27^

Issues related to difficulties with cut-off scores are also addressed in non-nephrology studies involving the Montreal Cognitive Assessment. Recently, a meta-analysis conducted in Canada,^28^ with versions of the MoCA in English, pointed out the problems regarding the sensitivity and specificity of the score, and demonstrated that the cut-off point of 23 provided for better classification accuracy (90%) and better balance between false and true positive issues (Youden index = 0.79). One of the few studies carried out with the Brazilian version of the MoCA, by Sarmento,^29^ suggested a cutoff point of 24 for MoCA in the Portuguese language, with a sensitivity of 70.0% and specificity of 62.5%. However, in this version, the author found low internal consistency of the instrument in its translation.

Thus, our analysis of the reviewed studies shows that there is still a need for more complete studies on scoring and the adaptation of the Montreal Cognitive Assessment for the different languages. It is important to consider the sociodemographic particularities of the different countries, especially regarding schooling and the translated version of the instrument to be used. In the clinical context, it is recommended that healthcare professionals seek studies that are more related to the context experienced by the patients for the interpretation of the results.

This analysis may be biased. The main risk of bias to which this study may be related is that of publication, since there is a greater propensity for authors to publish positive results obtained by their studies. The present review also presented some limitations. Only one of the authors was responsible for the selection and extraction of data from the articles. As a way to circumvent this limitation, the search criteria are presented as Supplementary Material in order to reduce the possible risk of bias. Another limitation is the non-performance of quality assessment of the studies through validated tools for observational studies. The selection of articles in Portuguese and English only can also be considered a limitation of the study; however, it seems to be of lesser force, since only two papers were not included because they were published in another language.

## Final considerations

MoCA has been used effectively in several stages and modalities of CKD treatment. However, there is no consensus in the literature regarding the cutoff point with better sensitivity and specificity for the detection of cognitive impairment. In summary, it is known that there is a high prevalence of cognitive impairment in the chronic renal patient population, present in the various stages of the disease, concerning the different treatment modalities, and in an irreversible way. It is considered that the articles analyzed provide an important basis for considering the need to adapt clinical practices performed by healthcare professionals, as well as to develop ideas to run new studies involving this population. Although further studies are required concerning the criterion used to identify cognitive impairment. The studies demonstrate the versatility of MoCA in the cognitive screening of chronic renal patients, providing results on the degree of impairment and functions with greater losses.

Based on the information gathered, it is important to motivate reflection and stimulate the creation of strategies by healthcare professionals concerning practices that may contribute to the prevention or retardation of the patients' cognitive impairment. It is believed that contributions on the subject should not be restricted to nephrologists, neurologists and neuropsychologists, but rather that all healthcare professionals can offer strategies, within their knowledge network, to confront and adapt this clinical picture.

## Supplementary material

The following supplementary material is available online:

PubMed search strategies

## References

[1] Condé SAL, Fernandes N, Santos FR, Chouab A, Mota MMEP, Bastos MG (2010). Declínio cognitivo, depressão e qualidade de vida em pacientes de
diferentes estágios da doença renal crônica. J Bras Nefrol.

[2] Paraizo Mde A, Almeida AL, Pires LA, Abrita RS, Crivellari MH, Pereira Bdos S (2016). Montreal Cognitive Assessment (MoCA) screening mild cognitive
impairment in patients with chronic kidney disease (CKD)
pre-dialysis. J Bras Nefrol.

[3] Lu R, Kiernan MC, Murray A, Rosner MH, Ronco C (2015). Kidney-brain crosstalk in the acute and chronic
setting. Nat Rev Nephrol.

[4] Matta SM, Moreira JM, Kummer AM, Barbosa IG, Teixeira AL, Silva ACS (2014). Cognitive alterations in chronic kidney disease: an
update. J Bras Nefrol.

[5] Foster R, Walker S, Brar R, Hiebert B, Komenda P, Rigatto C (2016). Cognitive Impairment in Advanced Chronic Kidney Disease: The
Canadian Frailty Observation and Interventions Trial. Am J Nephrol.

[6] Oliveira APB, Schmidt DB, Amatneeks TM, Santos JC, Cavallet LHR, Michel RB (2016). Quality of life in hemodialysis patients and the relationship
with mortality, hospitalizations and poor treatment
adherence. J Bras Nefrol.

[7] Nasreddine Z, Phillips N, Bédirian V, Charbonneau S, Whitehead V, Colllin I (2005). The Montreal Cognitive Assessment, MoCA: a brief screening tool
for mild cognitive impairment. J Am Geriatr Soc.

[8] Galvão TF, Pansani TSA, Harrad D (2015). Principais itens para relatar Revisões sistemáticas e
Meta-análises: A recomendação PRISMA. Epidemiol Serv Saúde.

[9] Iyasere O, Okai D, Brown E (2017). Cognitive function and advanced kidney disease: longitudinal
trends and impact on decision-making. Clin Kidney J.

[10] Shea YF, Lam MF, Lee MS, Mok MY, Lui SL, Yip TP (2016). Prevalence of Cognitive Impairment Among Peritoneal Dialysis
Patients, Impact on Peritonitis and Role of Assisted
Dialysis. Perit Dial Int.

[11] Otobe Y, Hiraki K, Hotta C, Nishizawa H, Izawa KP, Taki Y (2017). Mild cognitive impairment in older adults with pre-dialysis
patients with chronic kidney disease: Prevalence and association with
physical function. Nephrology (Carlton).

[12] Gupta A, Mahnken JD, Johnson DK, Thomas TS, Subramaniam D, Polshak T (2017). Prevalence and correlates of cognitive impairment in kidney
transplant recipients. BMC Nephrol.

[13] Lambert K, Mullan J, Mansfield K, Lonergan M (2017). A comparison of the extent and pattern of cognitive impairment
among predialysis, dialysis and transplant patients: A cross sectional study
from Australia. Nephrology (Carlton).

[14] Palmer ND, Sink KM, Smith SC, Xu J, Bowden DW, Hugenschmidt CE (2014). Kidney disease and cognitive function: African American-diabetes
heart study MIND. Am J Nephrol.

[15] Zheng K, Wang H, Hou B, You H, Yuan J, Luo K (2017). Malnutrition-inflammation is a risk factor for cerebral small
vessel diseases and cognitive decline in peritoneal dialysis patients: a
cross-sectional observational study. BMC Nephrol.

[16] Kim K, Kang GW, Woo J (2018). The Quality of Life of Hemodialysis Patients Is Affected Not Only
by Medical but also Psychosocial Factors: a Canonical Correlation
Study. J Korean Med Sci.

[17] Angermann S, Baumann M, Wassertheurer S, Mayer CC, Steubl D, Hauser C (2017). Pulse wave velocity is associated with cognitive impairment in
hemodialysis patients. Clin Sci.

[18] Kang GW, Lee IH, Ahn KS, Lee J, Ji Y, Woo J (2015). Clinical and psychosocial factors predicting health-related
quality of life in hemodialysis patients. Hemodial Int.

[19] Lee SH, Cho A, Min YK, Lee YK, Jung S (2018). Comparison of the montreal cognitive assessment and the
mini-mental state examination as screening tests in hemodialysis patients
without symptoms. Ren Fail.

[20] Gupta A, Thomas TS, Klein JA, Montgomery RN, Mahnken JD, Johnson DK (2018). Discrepancies between Perceived and Measured Cognition in Kidney
Transplant Recipients: Implications for Clinical Management. Nephron.

[21] Tiffin-Richards FE, Costa AS, Holschbach B, Frank RD, Vassiliadou A, Krüger T (2014). The Montreal Cognitive Assessment (MoCA) - a sensitive screening
instrument for detecting cognitive impairment in chronic hemodialysis
patients. PLoS One.

[22] Al Wakeel J, Al Harbi A, Bayoumi M, Al-Suwaida K, Al Ghonaim M, Mishkiry A (2012). Quality of life in hemodialysis and peritoneal dialysis patients
in Saudi Arabia. Ann Saudi Med.

[23] Faoro M, Hamdan AC (2017). Avaliação neuropsicológica da atenção concentrada, flexibilidade
cognitiva e velocidade de processamento no Diabetes Mellitus Tipo
2. Rev Neuropsicol Latinoam.

[24] Sociedade Brasileira de Diabetes (2007). Tratamento e acompanhamento do diabetes mellitus.

[25] Herrera-Añazco P, Benites-Zapata V, Hernandez AV, Mezones-Holguin E, Silveira-Chau M (2015). Mortality in patients with chronic kidney disease undergoing
hemodialysis in a public hospital of Peru. J Bras Nefrol.

[26] Nikic PM, Andric BR, Stojimirovic BB, Trbojevic-Stankovic J, Bukumiric Z (2014). Habitual coffee consumption enhances attention and vigilance in
hemodialysis patients. Biomed Res Int.

[27] Jha V, Garcia-Garcia G, Iseki K, Li Z, Naicker S, Plattner B (2013). Chronic kidney disease: global dimension and
perspectives. Lancet.

[28] Carson N, Leach L, Murphy KJ (2017). A re-examination of Montreal Cognitive Assessment (MoCA) cutoff
scores. Int J Geriatr Psychiatry.

[29] Sarmento ALR (2009). Apresentação e aplicabilidade da versão brasileira da MoCA (Montreal
Cognitive Assessment) para rastreio de Comprometimento Cognitivo
Leve.

