# Supplementary material for: Montreal Cognitive Assessment for cognitive assessment in chronic kidney disease: a systematic review
Source: J Bras Nefrol. 2019 Jan 24;41(1):112–23. doi: 10.1590/2175-8239-JBN-2018-0086 (PMC6534019; doi:10.1590/2175-8239-JBN-2018-0086)
Supplement: Supplementary file 1 [file 2175-8239-jbn-2018-0086-suppl1.pdf]

Braz. J. Nephrol. (J. Bras. Nefrol.)

Supplementary Material for "*Montreal Cognitive Assessment* for cognitive assessment in chronic kidney disease: a systematic review"

PubMed search strategies

Data Source	Search strategies
**PubMed**	("montreal"[All Fields] AND "cognitive"[All Fields] AND "assessment"[All Fields]) OR ("montreal cognitive assessment"[All Fields]) AND ("kidney diseases"[MeSH Terms] OR ("kidney"[All Fields] AND "diseases"[All Fields]) OR "kidney diseases"[All Fields] OR ("kidney"[All Fields] AND "disease"[All Fields]) OR "kidney disease"[All Fields])
